# Active Contour Model Coupling with Higher Order Diffusion for Medical Image Segmentation

**DOI:** 10.1155/2014/237648

**Published:** 2014-03-02

**Authors:** Guodong Wang, Jie Xu, Qian Dong, Zhenkuan Pan

**Affiliations:** ^1^College of Information Engineering, Qingdao University, Qingdao 266071, China; ^2^College of Physics Science, Qingdao University, Qingdao 266071, China; ^3^The Affiliated Hospital of Medical College, Qingdao University, Qingdao 266003, China

## Abstract

Active contour models are very popular in image segmentation. Different features such as mean gray and variance are selected for different purpose. But for image with intensity inhomogeneities, there are no features for segmentation using the active contour model. The images with intensity inhomogeneities often occurred in real world especially in medical images. To deal with the difficulties raised in image segmentation with intensity inhomogeneities, a new active contour model with higher-order diffusion method is proposed. With the addition of gradient and Laplace information, the active contour model can converge to the edge of the image even with the intensity inhomogeneities. Because of the introduction of Laplace information, the difference scheme becomes more difficult. To enhance the efficiency of the segmentation, the fast Split Bregman algorithm is designed for the segmentation implementation. The performance of our method is demonstrated through numerical experiments of some medical image segmentations with intensity inhomogeneities.

## 1. Introduction 

Medical images are popular in real world because they give intuitive expression. With the development of image processing, computer-aided diagnosis are more important with the ever increasing images. Image segmentation is often the first step in computer-aided diagnosis. But medical images are often with intensity inhomogeneities; so it is a hard work to segment in such images as angiogram and MR bladder images.

The active contour model has been increasingly applied to image segmentation in the past decade, because it provides very good frameworks of variational image segmentations. Chan-Vese (CV) model [1] is the most popular active contour model for image segmentation based on the feature of mean gray value of different regions. Chen et al. [2] use variance feature to carry out the segmentation. The variance information can solve the question of different areas with same mean gray value and different variance. The MAP (maximum a posterior) is an efficient region descriptor for the segmentation task. Zhu [3], Paragios and Deriche [4], and Rousson and Deriche [5] approximated the MAP of the given image by a mixture of Gaussians. The features deduced by MAP method are very popular in images with different probability distributions. Sarti et al. [6] deduced using variance information by MAP of Rayleigh distribution.

The above methods are the key development in active contour models. But they are a failure in image segmentation with intensity inhomogeneities, because there are no features that can be deduced for segmentation using the active contour model. The images with intensity inhomogeneities often occurred in real world especially in medical images. Li et al. [7] are the first researchers to deal with this question using region scalable fitting energy. Then, they [8] also extend the method to MRI. Qian et al. [9] use a domain kernel function and an edge indicator function for medical image segmentation. He incorporates the information of different areas and edges for the segmentation.

Pan et al. [10] proposed a segmentation method based on global variables difference. The method is proposed for medical images with complex topological structure, strong contrast, and low noise characteristics. It makes full use of the image area information, builds an energy model, and uses variation gradient information to establish a global energy model to get the minimization value. Yao and Cheng [11] use adjustable method for medical image segmentation. They combine active contour model with diffusion filter for multiobject segmentation of the noisy image. A target adaptive scheme is designed for adjusting the model to solving a particular image processing task.

All the methods mentioned above are region-based models aiming to identify each region of interest by using a certain region descriptor to guide the motion of the active contour. For images with intensity inhomogeneity, region descriptor is hard to find. There is much important information such as image gradient and Laplace that are ignored in active contour model. For medical images, intensity inhomogeneity is usually due to technical limitations or artifacts introduced by the object being imaged. The edges are blurred and the gradients are weak. So incorporate edge information alone is not enough, we also incorporate higher order diffusion term into the active contour model aiding segmentation.

In this paper, we propose a new active contour model with gradient and higher-order information of the image. The model can also be deemed as active contour model coupling with high-order diffusion, diffusion, because the introduction of Laplace information and the difference scheme becomes more difficult. To enhance the efficiency of the segmentation, the fast Split Bregman algorithm is designed for the segmentation implementation.

The organization of this paper goes as follows. In Section 2, we will introduce the active contour and high-order diffusion related methods briefly. Then the new active model with high-order diffusion method is proposed in Section 3. Then some numerical examples are shown in Section 4.  Section 5 is the concluding remarks.

## 2. Higher Diffusion and Active Contour Model

Generalized Perona-Malik equation for image restoration [12] was the first paper that introduced and implemented high-order anisotropic diffusion partial differential equations (PDEs) for image analysis. There are also many other high-order PDEs that have been already applied to image processing [13, 14]. They achieved great success in various image processing applications. The models are proposed for staircase effect reduction. Incorporating Laplace term, higher-order diffusion can use more information near the edges. In this paper, we select the total variation with higher-order diffusion because of the simplicity.

The total variation model with higher-order diffusion is depicted as:

(1) E(u)=12∫Ω(u−f)2dx dy+λ1∫Ω|∇u|dx dy+λ2∫Ω|Δu|dx dy,

where *Ω* is the image domain,  *u*  is the denoised image, and *f* is the noisy image.

The active contour model using mean gray value is depicted as:

(2) arg min⁡ϕ∈[0,1]{∫Ω|∇ϕ|dx dy      +∫Ω(α1|f−u1|2ϕ+α2|f−u2|2(1−ϕ))dx dy},

where *ϕ* is the level set function and *u*_1_ and *u*_2_ are the mean gray value of different areas. This contour model is a global convex model proposed by Bresson et al. [15]. The proposed model is a global minimization problem due to convex set *ϕ* ∈ [0,1]. Bresson transformed the original active contour model to a convex minimization problem by relaxing *ϕ* ∈ {0,1} to *ϕ* ∈ [0,1] and showed that the characteristic function is the global minimizer. This model is based on mean gray value and it has drawbacks with different usages.

For medical images we incorporate the edges and Laplace information into active contour model for dealing with intensity inhomogeneities. The new model has the abilities of both (1) and (2). The new model is depicted in the next section.

## 3. Active Contour Model Coupling with Higher-Order Diffusion

It is hard to segment medical images because the edges are always weak and the images are also noisy. We couple the higher-order diffusion model with active contour model for medical images segmentation. The new model that couples higher-order diffusion with active contour model is

(3) arg min⁡ϕ∈[0,1]{∫Ω|∇ϕ|dx dy+∫Ω((α1|f−u1|2+β1|∇u1|+γ1|Δu1|)ϕ+(α2|f−u2|2+β2|∇u2|+γ2|Δu2|)(1−ϕ))dx dy},

where *Ω* is the image domain, *α*_1_,  *β*_1_,  *γ*_1_,  *α*_2_,  *β*_2_,  *γ*_2_ are the positive parameters, *f* is the original image, *u*_1_ and *u*_2_ are the mean values in different image parts, and *ϕ* is a standard level set function.

We use the form of (3) mainly because the image features in different areas are not always the same. This is a natural extension of Chen and Vese [1] and Vese and Chen [16] model for piecewise constant or smooth model. We can get the solution using alternating iteration. Because there are gradient and Laplace information, straightforward solving of (3) by introducing forth-order term of difference scheme is a hard work. The Split Bregman method [17, 18] is introduced for the easy implementation of the model.

We introduce the auxiliary variables $\overset{⃑}{w_{1}}={\left(w_{11},w_{12}\right)}^{T}$,  *w*_2_ and Bregman iteration parameters$\;\;\overset{⃑}{b}={\left(b_{1},b_{2}\right)}^{T}$, when the following energy function gets its minimization, $\overset{⃑}{w_{1}}\approx \nabla u,\;\;w_{2}\approx \Delta u$.

Equation (3) can be divided into the following three sub-problems of minimization:

Fix  *ϕ*  and  *u*_2_, the energy function of (3) became the following form:

(4) (u1k+1,w1⃑k+1,w2)=arg min⁡w1⃑,u1{∫Ω(β1|w1⃑|+γ1|w2|+α1|f−u1|2+μ12(w1⃑−∇u1−b1⃑k)2+θ12(w2−∇·w1⃑)2)ϕ dx dy},

where ${\overset{⃑}{w_{1}}}^{k+1}=\nabla {u_{1}}^{k+1}$,$\;\;{\overset{⃑}{b_{1}}}^{k+1}={\overset{⃑}{b_{1}}}^{k}+\nabla {u_{1}}^{k+1}-{\overset{⃑}{w_{1}}}^{k+1}$,$\;\;w_{2}=\nabla \cdot \overset{⃑}{w_{1}}$,  *μ*_1_, and  *θ*_1_  are the positive parameters.

The energy function of *u*_1_ is

(5) $$\begin{array}{c} E\left(u_{1}\right)=\int_{\Omega }\left(\alpha_{1}{\left|f-u_{1}\right|}^{2}+\frac{\mu_{1}}{2}{\left(\overset{⃑}{w_{1}}-\nabla u_{1}-{\overset{⃑}{b_{1}}}^{k}\right)}^{2}\right)\phi \;dx\;dy. \end{array}$$

The Eular-Lagrange equation is

(6) $$\begin{array}{c} 2\alpha_{1}\left(u_{1}-f\right)\phi -\mu_{1}\nabla \cdot \left(\phi \left(\nabla {u_{1}}^{k+1}+{\overset{⃑}{b_{1}}}^{k}-{\overset{⃑}{w_{1}}}^{k}\right)\right)=0. \end{array}$$

The discretion of *u*_1_ is

(7) u1=1(2α1+4μ1)×(2α1f+μ1(u1,i,j+1k+u1,i,j−1k            +u1,i+1,jk+u1,i−1,jk            +∇·(b1⃑k−w1⃑k))).

The energy function of $\overset{⃑}{w_{1}}$ is

(8) E(w1⃑)=∫Ω(β1|w1⃑|+μ12(w1⃑−∇u1−b1⃑k)2   +θ12(w2−∇·w1⃑)2)ϕ dx dy.

Using Eular-Lagrange equation, we can get

(9) w1⃑k+1=∇uk+1+bk+1+θ1μ1∇(∇·w1⃑−w2)−β1μ1w1⃑k+1|w1⃑k+1|.

By wavelet soft threshold

(10) w1⃑k+1=max⁡(|∇u1k+1+b1⃑k+θ1μ1∇(∇·w1⃑−w2)|−β1μ1,0)×∇u1k+1+b1⃑k+(θ1/μ1)∇(∇·w1⃑−w2)|∇u1k+1+b1⃑k+(θ1/μ1)∇(∇·w1⃑−w2)|.

The energy function of *w*_2_ is

(11) $$\begin{array}{c} E\left(w_{2}\right)=\int_{\Omega }\left(\gamma_{1}\left|w_{2}\right|+\frac{\theta_{1}}{2}{\left(w_{2}-\nabla \cdot \overset{⃑}{w_{1}}\right)}^{2}\right)\phi \;dx\;dy. \end{array}$$

Using Eular-Lagrange equation, we can get

(12) $$\begin{array}{c} {w_{2}}^{k+1}=\nabla \cdot {w_{1}}^{k+1}-\frac{\gamma_{1}}{\theta_{1}}\frac{{w_{2}}^{k+1}}{\left|{w_{2}}^{k+1}\right|}. \end{array}$$

By wavelet soft threshold,

(13) $$\begin{array}{c} {w_{2}}^{k+1}=\max\left(\left|\nabla \cdot {w_{1}}^{k+1}\right|-\frac{\gamma_{1}}{\theta_{1}},0\right)\frac{\nabla {w_{2}}^{k+1}}{\left|\nabla {w_{2}}^{k+1}\right|}. \end{array}$$

For solving *u*_2_, the procedure is the same as solving *u*_1_.

For convenience, we write

(14) R(u1,u2)=(α1|f−u1|2+β1|∇u1|+γ1|Δu1|)−(α2|f−u2|2+β2|∇u2|+γ2|Δu2|).

The energy function can be rewritten as:

(15) $$\begin{array}{c} \underset{\phi \in \left[0,1\right]}{\arg\;\min}\left\{E\left(\phi \right)=\int_{\Omega }\left|\nabla \phi \right|dx\;dy+\int_{\Omega }R\left(u_{1},u_{2}\right)\phi \;dx\;dy\right\}. \end{array}$$

For solving *ϕ*, we also use the Split Bregman method by introducing $\overset{⃑}{v}=\nabla \phi$.

Then the energy function of (14) is transformed as:

(16) (ϕk+1,v⃑k+1) =arg min⁡d⃑,ϕ∈[0,1]{∫Ω|v⃑|dx dy         +∫ΩR(u1,u2)ϕ dx dy         +λ2∫Ω(v⃑−∇ϕ−d⃑k)2dx dy},

where ${\overset{⃑}{d}}^{k+1}={\overset{⃑}{d}}^{k}+\nabla \phi^{k+1}-{\overset{⃑}{v}}^{k+1}$, *λ* is the positive parameter.

Fixing $\overset{⃑}{v}$ for solving *ϕ*:

(17) $$\begin{array}{c} R\left(u_{1},u_{2}\right)-\lambda \left(\Delta \phi^{k+1}+\nabla \cdot {\overset{⃑}{d}}^{k}-\nabla \cdot {\overset{⃑}{v}}^{k}\right)=0. \end{array}$$

Using gradient descent method,

(18) $$\begin{array}{c} \frac{d\phi }{dt}=\lambda \left(\Delta \phi^{k+1}+\nabla \cdot {\overset{⃑}{d}}^{k}-\nabla \cdot {\overset{⃑}{v}}^{k}\right)-R\left(u_{1},u_{2}\right). \end{array}$$

After discretion,

(19) ϕk+1 =(11+4λdt)dt  ×(λ(ϕi,j−1k+ϕi,j+1k+ϕi−1,jk+ϕi+1,jk+∇·d⃑k−∇·v⃑k)      −R(u1,u2)).

To ensure that *ϕ* ∈ [0,1], so in every step we use the following function:

(20) $$\begin{array}{c} \phi^{k+1}=\min\left(\max\left(\phi^{k+1},0\right),1\right). \end{array}$$

Fix *ϕ* for solving $\overset{⃑}{v}$, we can get

(21) $$\begin{array}{c} {\overset{⃑}{v}}^{k+1}=\max\left(\left|\nabla \phi^{k+1}+{\overset{⃑}{d}}^{k}\right|-\frac{1}{\lambda },0\right)\frac{\nabla \phi^{k+1}+{\overset{⃑}{d}}^{k}}{\left|\nabla \phi^{k+1}+{\overset{⃑}{d}}^{k}\right|}. \end{array}$$

In the end of calculation, we set

(22) $$\begin{array}{c} \phi \left(x\right)=\{\begin{array}{cc} 0 & \phi \left(x\right)\geq \text{ th }\\ 1 & \phi \left(x\right)<\text{th}, \end{array} \end{array}$$

where th is the preset threshold. Thus the foreground and the background can be separated. Due to *ϕ* ∈ [0,1], our new model is also globally convex. That is to say, the position of *ϕ* need not be initialized.

## 4. Numerical Experiments

To verify the effect of our proposed method, we test our method on a variety of real images with intensity inhomogeneity. We compare our method with CV model [1] and mean shift algorithm [19] because they are highly cited and compared in image segmentation. The CV model which is used in our experiment for comparison is also global convex [15]. For comparison with the mean shift algorithm, we used the software EDISON which is based on a fast implementation of the mean shift algorithm using a speed-up scheme described in [19–21].

**Figure 1:**
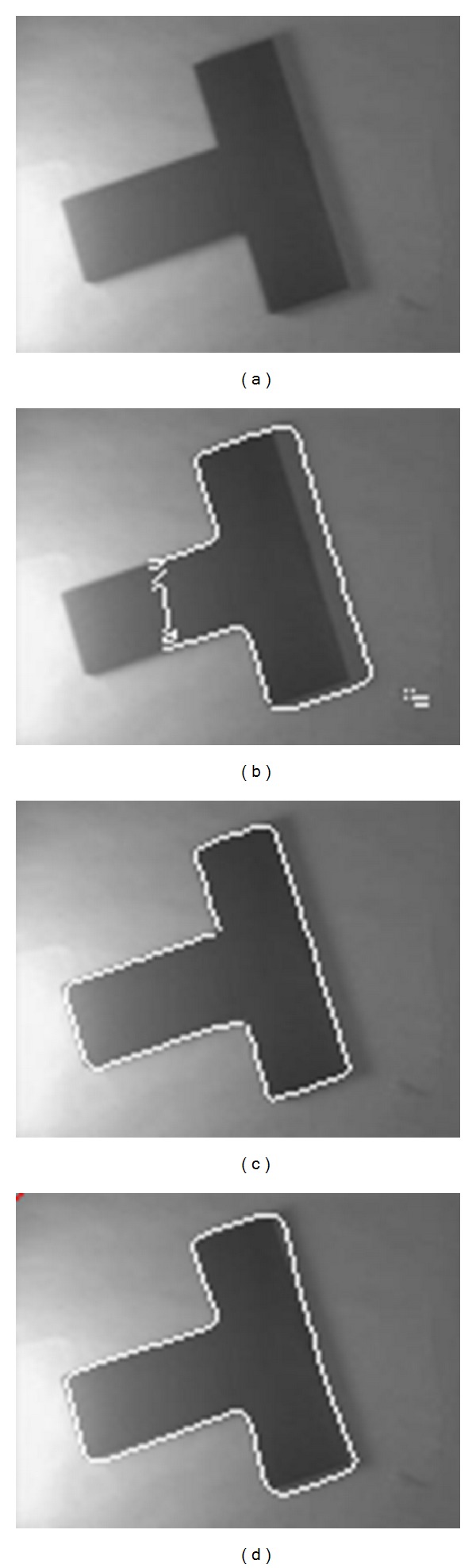
Image for segmentation. (a) Original image. (b) Result using CV model. (c) Result using mean shift method. (d) Segmentation result using proposed method.

**Figure 2:**
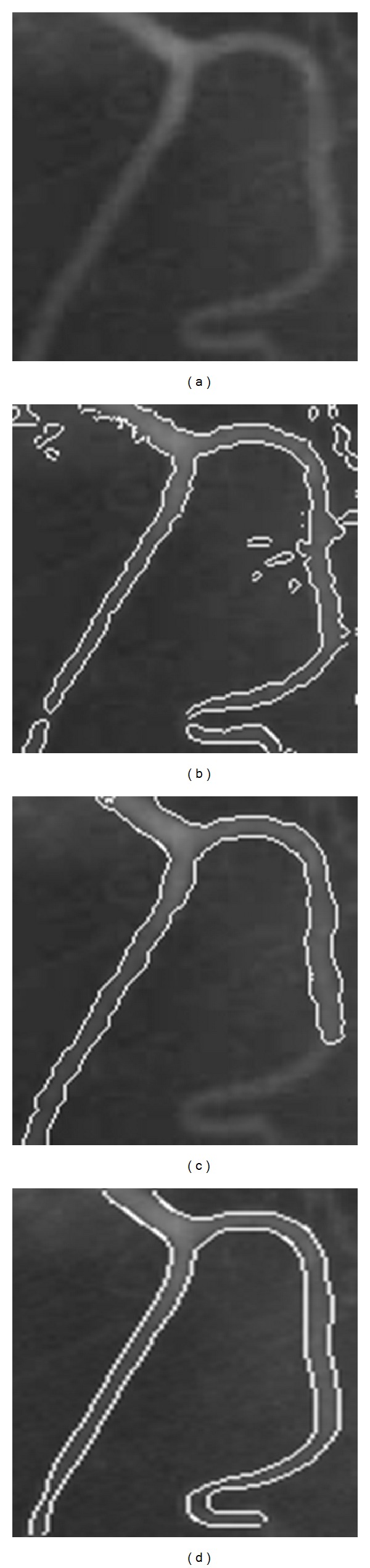
Image for segmentation. (a) Original image. (b) Result using CV model. (c) Result using mean shift method. (d) Segmentation result using proposed method.

**Figure 3:**
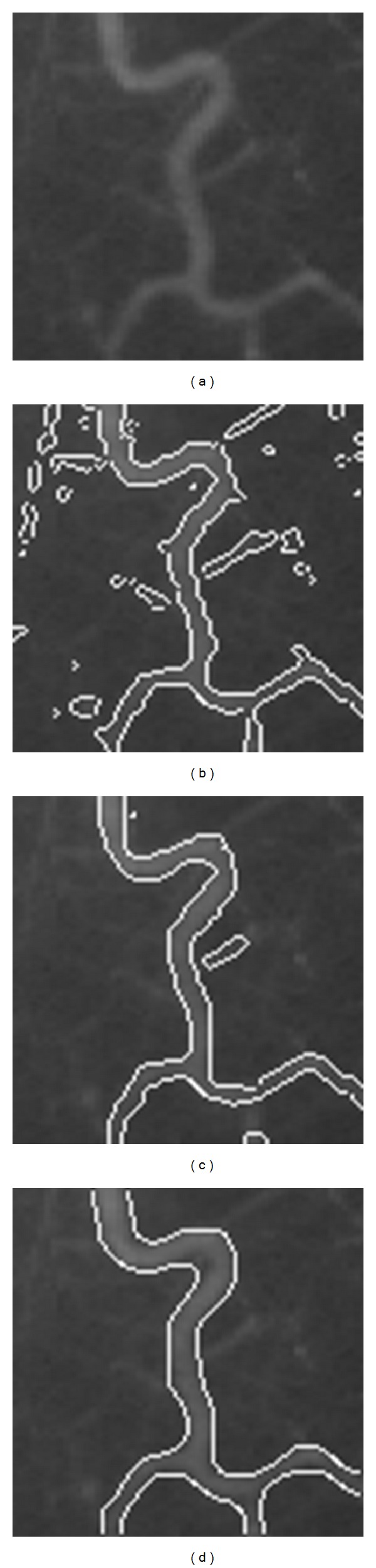
Image for segmentation. (a) Original image. (b) Segmentation result using CV model. (c) Segmentation result using mean shift method. (d) Segmentation result using proposed method.

**Figure 4:**
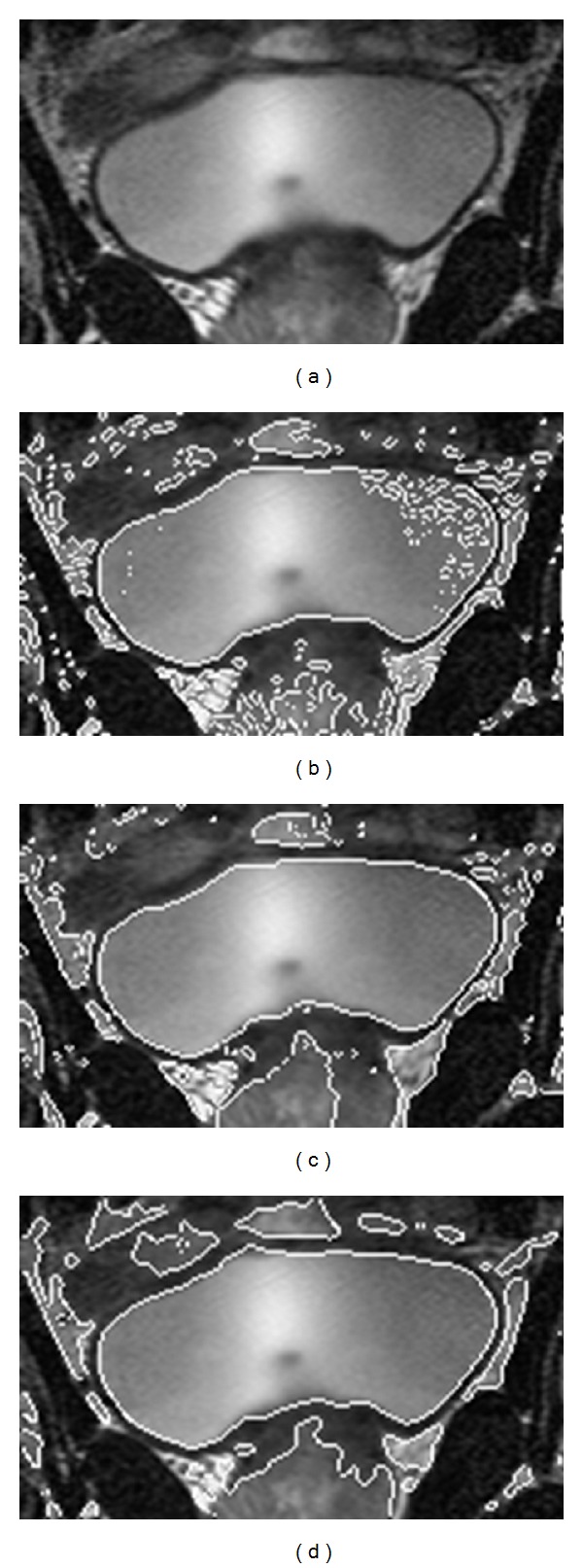
Experiments for an MR image of bladder. (a) Original image. (b) Segmentation result using CV model. (c) Segmentation result using mean shift method. (d) Segmentation result using proposed method.

Figure 1 shows the results for a real image of a T-shaped object with different lighting intensity. Figures 2 and 3 are two experiments on X-ray images of vessels. The images are noisy with intensity inhomogeneity.  Figure 4 is a test of MR image of blades with the comparison of CV model and mean shift method. All of them are selected because they are typical images with intensity inhomogeneity. The CV model and mean shift method cannot get the proper results while our model can get the right results. The shadow of the object is the key element causing wrong segmentation of tradition methods. However, our model can separate the shadow and the object.

The vessel images in Figures 2 and 3 fail to be segmented using traditional methods because of the intensity inhomogeneity. But using our model, the boundary of the object of interest (the bladder) is extracted very well. In these two images, parts of the vessel boundaries are quite weak; our method can still segment them well. Satisfactory segmentation results have been obtained for these challenging images. Our method successfully extracts the object boundaries for these two images.

Figure 4 is the segmentation results of an MR image of bladder. The result of mean shift algorithm for the first image is similar to that of our method, showing certain ability of the mean shift algorithm in handling intensity inhomogeneity. However, for the two-vessel image, a small portion of the vessel is missing. For segmenting the MR bladder image, the result is not so perfect for surrounding organs. We notice that the segmentation result of mean shift algorithm is somewhat sensitive to the choice of two major parameters: spatial bandwidth and range bandwidth. We have tweaked these two parameters and other minor parameters for the best segmentation results for these four images.

## 5. Conclusion

In this paper, we present a new active contour model for medical image segmentation. The proposed model incorporates gradient and Laplace information. It can segment images with intensity inhomogeneity and has desirable performance for images with weak object boundaries. Experimental results have demonstrated the advantages of our method over several well-known methods for image segmentation.
